# Non-Immune Binding of Human IgG to M-Related Proteins Confers Resistance to Phagocytosis of Group A Streptococci in Blood

**DOI:** 10.1371/journal.pone.0078719

**Published:** 2013-10-25

**Authors:** Harry S. Courtney, Yi Li

**Affiliations:** Veterans Affairs Medical Center and the Department of Medicine, University of Tennessee Health Science Center, Memphis, Tennessee, United States of America; Centers for Disease Control & Prevention, United States of America

## Abstract

The non-immune binding of immunoglobulins by bacteria is thought to contribute to the pathogenesis of infections. M-related proteins (Mrp) are group A streptococcal (GAS) receptors for immunoglobulins, but it is not known if this binding has any impact on virulence. To further investigate the binding of immunoglobulins to Mrp, we engineered mutants of an M type 4 strain of GAS by inactivating the genes for *mrp*, *emm*, *enn*, *sof*, and *sfbX* and tested these mutants in IgG-binding assays. Inactivation of *mrp* dramatically decreased the binding of human IgG, whereas inactivation of *emm*, *enn*, *sof*, and *sfbx* had only minor effects, indicating that Mrp is a major IgG-binding protein. Binding of human immunoglobulins to a purified, recombinant form of Mrp indicated that it selectively binds to the Fc domain of human IgG, but not IgA or IgM and that it preferentially bound subclasses IgG_1_>IgG_4_>IgG_2_>IgG_3_. Recombinant proteins encompassing different regions of Mrp were engineered and used to map its IgG-binding domain to its A-repeat region and a recombinant protein with 3 A-repeats was a better inhibitor of IgG binding than one with a single A-repeat. A GAS mutant expressing Mrp with an in-frame deletion of DNA encoding the A-repeats had a dramatically reduced ability to bind human IgG and to grow in human blood. Mrp exhibited host specificity in binding IgG; human IgG was the best inhibitor of the binding of IgG followed by pig, horse, monkey, and rabbit IgG. IgG from goat, mouse, rat, cow, donkey, chicken, and guinea pig were poor inhibitors of binding. These findings indicate that Mrp preferentially binds human IgG and that this binding contributes to the ability of GAS to resist phagocytosis and may be a factor in the restriction of GAS infections to the human host.

## Introduction

The group A streptococcus, *Streptococcus pyogenes*, is an important human pathogen that is estimated to be the ninth leading cause of deaths due to microbial infections worldwide [1]. Members of the M protein family are key virulence factors that contribute to the pathogenesis of *S. pyogenes* infections and their binding of blood proteins, such as complement regulatory proteins, plasminogen, albumin, fibrinogen, and immunoglobulins, is thought to contribute to pathogenesis [2-14].

**Figure 1:**
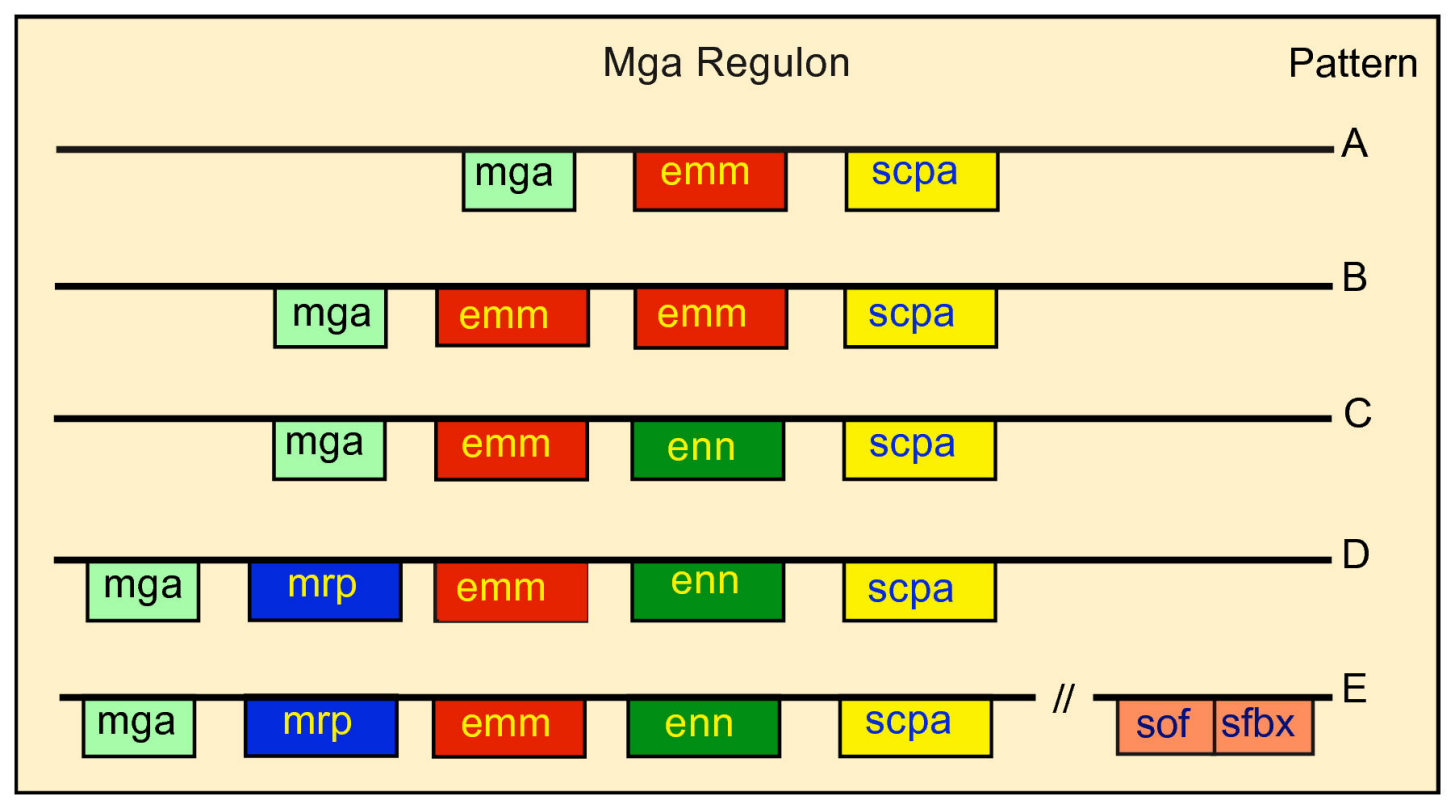
Variations of the Mga regulon. Mga (multigene activator) is a positive regulator of a number of streptococcal genes. The most prominent of these are the family of M proteins whose genes are tandemly linked. sof (serum opacity factor) and *sfbx* (streptococcal fibronectin binding protein x) are bicistronic and are also regulated by Mga, but are located some distance away. *emm* encodes for M protein, *mrp* encodes M-related proteins, *enn* encodes an M-like protein that binds IgA, and *scpa* encodes a C5a peptidase. Some serotypes contain only *mga*, *emm*, and *scpa* (pattern A). Other serotypes contain one or more of the remaining genes (patterns B–E). The figure is derived from the data and classification scheme of Bessen and co-workers [29,30] and is copied with permission from [31,32].

The M protein family is composed of M protein (Emm), M-related protein (Mrp), and an M-like protein (Enn), which are part of the Mga regulon (Figure 1). The components of the Mga regulon can vary depending upon the serotype. Some serotypes express only Emm (Pattern A), whereas other serotypes express Emm, Mrp and/or Enn (Figure 1). Interestingly, it appears that some of the functions of Emm in those serotypes that express only Emm (pattern A) are shifted to other members of the M protein family in those serotypes that express Mrp and Enn (patterns C, D, and E). For example, Emm binds fibrinogen in pattern A serotypes whereas Mrp is the major fibrinogen-binding protein in pattern D and E serotypes [3,5,7,11]. 

Infections caused by *S. pyogenes* are almost entirely restricted to humans, but the molecular basis for this host preference is poorly understood. Plasminogen binding has been linked to host specificity of group A streptococcal infections [15], and the ability of *S. pyogenes* to selectively bind immunoglobulins from certain species is thought to contribute to this host specificity and to virulence. Mrp is a major surface protein of *S. pyogenes* that has been shown to bind human IgG [16-18], but there is no evidence indicating that this binding has a role in virulence. Herein, we present our findings that support a role for Mrp-IgG interactions as a factor contributing to virulence and host specificity of *S. pyogenes*. 

## Materials and Methods

### Reagents

Peroxidase-labeled, human IgG, IgM, IgA and the unlabeled F(ab’)_2_ and Fc fragments of human IgG were purchased from Jackson ImmunoResearch Labs, Inc. (West Grove, PA). Unlabeled animal IgG and sera were obtained from Sigma Aldrich (St. Louis, MO) and Innovative Research (Novi, MI). Human IgG_1_, IgG_2_, IgG_3_ and IgG_4_ were obtained from Athens Research & Technology, Inc. (Athens, GA). Human, plasminogen-free fibrinogen was purchased from Calbiochem (La Jolla, CA) and biotinylated by the method of Bayer et al. [19]. Neutravidin-peroxidase was obtained from Pierce (Rockford, IL). The preparation of rabbit antiserum against a synthetic peptide copying amino acid residues 3-17 of Mrp4 (anti-sMrp4(3-17)) and residues 1-30 of Emm4 (anti-sEmm4(1-30)) was previously described [5]. 

### Bacterial strains, mutants and growth conditions

The construction of mutants utilizing the parental strain M type 4 *S. pyogenes* (SP4) was previously described [5]. These consisted of MP4, an Mrp-negative mutant; AR4, an Emm-negative mutant; EP4, an Enn-negative mutant; SF4, a SOF-negative mutant; and DS4, an Sof-negative and Sfbx-negative mutant. The mutant SP4ΔA, which expresses Mrp in which the A-repeats were deleted in-frame, was constructed by cutting the desired sequences from the pTrcHis vector that contained an insert of rMrpΔA DNA (see below, cloning of rMrp for details) and ligating the insert into pG+Host9, a temperature-sensitive shuttle vector generously provided by E. Maguin [20]. The vector was then introduced into SP4 via allelic exchange and a mutant expressing Mrp with an in-frame deletion of the A-repeats was selected by previously described methods [5]. The strains were grown overnight at 37°C in Todd-Hewitt broth supplemented with 1% yeast extract (THY) unless indicated otherwise.

### Cloning, expression, and purification of recombinant Mrp

 DNA encoding the desired sequences of Mrp4 were amplified by PCR, ligated into pTrcHis, introduced into *Escherichia coli* Top10, expressed as histidine fusion products, and purified by metal affinity chromatography as previously described [5]. The recombinant proteins consisted of rMrp(1-328), rMrp(150-255), rMrp(150-185), rMrp(256-328), rMrp(1-184), rMrp(97-197). The numbers in each case indicate the amino acid residues that are spanned in the mature form of Mrp4. rMrpΔA, in which the DNA encoding the A-repeats was deleted in-frame, was constructed as follows. The region of *mrp* upstream of the A-repeats and the region of *mrp* immediately downstream of the A-repeats were amplified by PCR, sequentially inserted and ligated in-frame into the pTrcHis vector, then expressed and purified as above.

### Enzyme-linked immunoassays for binding of IgG and fibrinogen to M type 4 *S. pyogenes* and its mutants

The wild type strain SP4 and the indicated mutants were grown overnight in THY, washed in phosphate buffered saline (PBS), and adjusted to an OD_530_ of 0.4 in PBS. Microtiter wells were coated with 100 µl of the streptococcal suspension for 30 min at 37 °C, then washed and blocked with 1% BSA in PBS. The wells were then reacted with various concentrations of peroxidase-labeled, human IgG or biotinylated, human fibrinogen for 30 min at 37 °C. The wells treated with peroxidase-labeled, human IgG were then washed and 100 µl of the substrate tetramethylbenzidine (TMB) was added. For wells treated with biotinylated fibrinogen, the wells were incubated with 1 µg/ml of Neutravidin-peroxidase for 30 min at 37°C, then washed and the TMB substrate added. The absorbance at 650 nm was recorded after color development. Blanks consisted of wells coated with BSA instead of streptococci and then treated as described above. Assays were done in quadruplicate.

### Enzyme-linked immunoassays for expression of surface proteins in SP4 and SP4∆A

The parental strain SP4 and its mutant SP4ΔA were grown overnight in THY, washed in PBS and used to coat microtiter wells as described above. The coated wells were reacted for 30 min at 37°C with rabbit anti-sMrp4(3-17) and anti-sEmm4(1-30) diluted 1:500 in Tris-saline-BSA (0.05 M Tris-HCl, 0.15 M NaCl, 1 mg/ml bovine serum albumin, pH 7.4) containing 10% pig serum to block non-immune binding of immunoglobulins. The wells were then washed and reacted with a 1:2000 dilution of peroxidase-labeled, goat anti-rabbit IgG diluted in Tris-saline-BSA. After 30 min at 37°C the wells were washed, 100 µl of TMB substrate added and the absorbance at 650 nm was recorded after color development. Control wells were reacted under the same conditions with normal rabbit serum to determine the degree of non-specific binding. Assays were done in quadruplicate.

### Assays for effect of animal sera and animal IgG on the binding of human IgG to Mrp

Microtiter wells were coated with 100 µl of 2.5 µg/ml of rMrp(1-328) in sodium bicarbonate (pH 9.5) for 1 hour at 37°C. The wells were washed and blocked with 1% BSA in PBS. Afterwards, 100 µl of 1 µg/ml of peroxidase-conjugated human IgG in a 10% solution of the indicated animal serum or in various concentrations of animal IgG diluted in Tris-saline-BSA were added to the appropriate wells. The positive control consisted of 1 µg/ml of peroxidase-conjugate human IgG in Tris-saline-BSA. Wells coated with BSA and treated as above served as negative controls. The wells were incubated at 37°C for 30 min, washed with Tris-saline, and 100 µl of the TMB substrate added. The absorbance at 650 nm was recorded after color development. Percent inhibition was calculated using the formula: % inhibition = [1- (A_650_ of animal serum (or animal IgG)/A_650_ of positive control)] x 100. Assays were done in quadruplicate. 

### Competitive binding assays for effect of IgG subclasses, F(ab’)_2_ and Fc regions of IgG on binding of IgG to Mrp

Microtiter wells were coated with 2.5 µg/ml of rMrp(1-328) and then blocked with 1 mg/ml BSA in PBS. A 100 µl aliquot of a solution of 1 µg/ml peroxidase-conjugated human IgG and various concentrations of human IgG subclasses 1-4 or the F(ab’)_2_ or the Fc fragment of human IgG in Tris-saline-BSA + 0.05% Tween-20 was added to the wells and incubated at 37°C for 30 min. The wells were then washed, TMB substrate added and the absorbance at 650 nm recorded after color development. Negative controls consisted of wells coated with BSA and treated as described above. Positive controls consisted of Mrp-coated wells treated with 1 µg/ml peroxidase-conjugated human IgG in Tris-saline-BSA + 0.05% Tween-20. Percentage of inhibition was calculated using the formula: % inhibition = [1- (A_650_ of binding of IgG in solutions containing the F(ab’)_2_ (or Fc, or IgG_1-4_)/A_650_ of positive control)] x 100. Assays were done in triplicate.

### Enzyme-linked immunoassays to map the IgG-binding domain of Mrp

To map the IgG-binding domains of Mrp4, microtiter wells were coated with 10 µg/ml of the indicated recombinant peptides of Mrp for 30 min at 37 °C and then blocked with 1% BSA in PBS for 30 min at 37 °C. Various concentrations of peroxidase-labeled human IgG in Tris-saline-BSA were then added to the wells and incubated for 30 min at 37 °C. After washing, the TMB substrate was added, and the absorbance at 650 nm was recorded after color development. Negative controls consisted of wells coated with BSA and treated as described above. Control assays using antiserum against the polyhistidine tag indicated that all of the histidine-tagged recombinant proteins of Mrp coated the microtiter wells to a similar degree. Assays were done in triplicate.

### Competitive binding assays comparing a single A-repeat to multiple A-repeats of Mrp as inhibitors of binding of human IgG to Mrp

Microtiter wells were coated with rMrp(1-328), blocked with BSA, and then reacted with peroxidase-labeled, human IgG in various concentrations of rMrp(150-185) (a single A-repeat) or rMrp(150-255) (three A-repeats) for 30 min at 37°C. The wells were then washed, the TMB substrate added and the absorbance at 650 nm recorded after color development. Assays were done in triplicate. 

### Streptococcal growth in human blood

 For growth assays in human blood, SP4 and its mutant SP4ΔA, were grown at 37°C in Todd-Hewitt broth supplemented with 1% yeast extract (THY) to an OD_530_ of 0.08 and diluted 1:10,000 in THY. A 50 µl aliquot of this dilution was added to tubes containing 450 µl of heparinized, human blood. The mixtures were rotated for 3 hours at 37 °C and the number of CFU were determined by plating dilutions on blood agar plates. The experiments were designed to provide an inoculum in the range of 100 to 300 CFU and the number of CFU in the inoculum was determined by plating on blood agar. The blood of donors was screened to ensure that their blood did not contain antibodies that could opsonize *S. pyogenes* and alter their growth in blood.

### Ethics statement

 All blood donors signed a written informed consent and the study and the consent form were approved by the Institutional Review Board of the University of Tennessee Health Science Center. 

## Results

### Role of Mrp in binding IgG on the streptococcal surface

**Figure 2:**
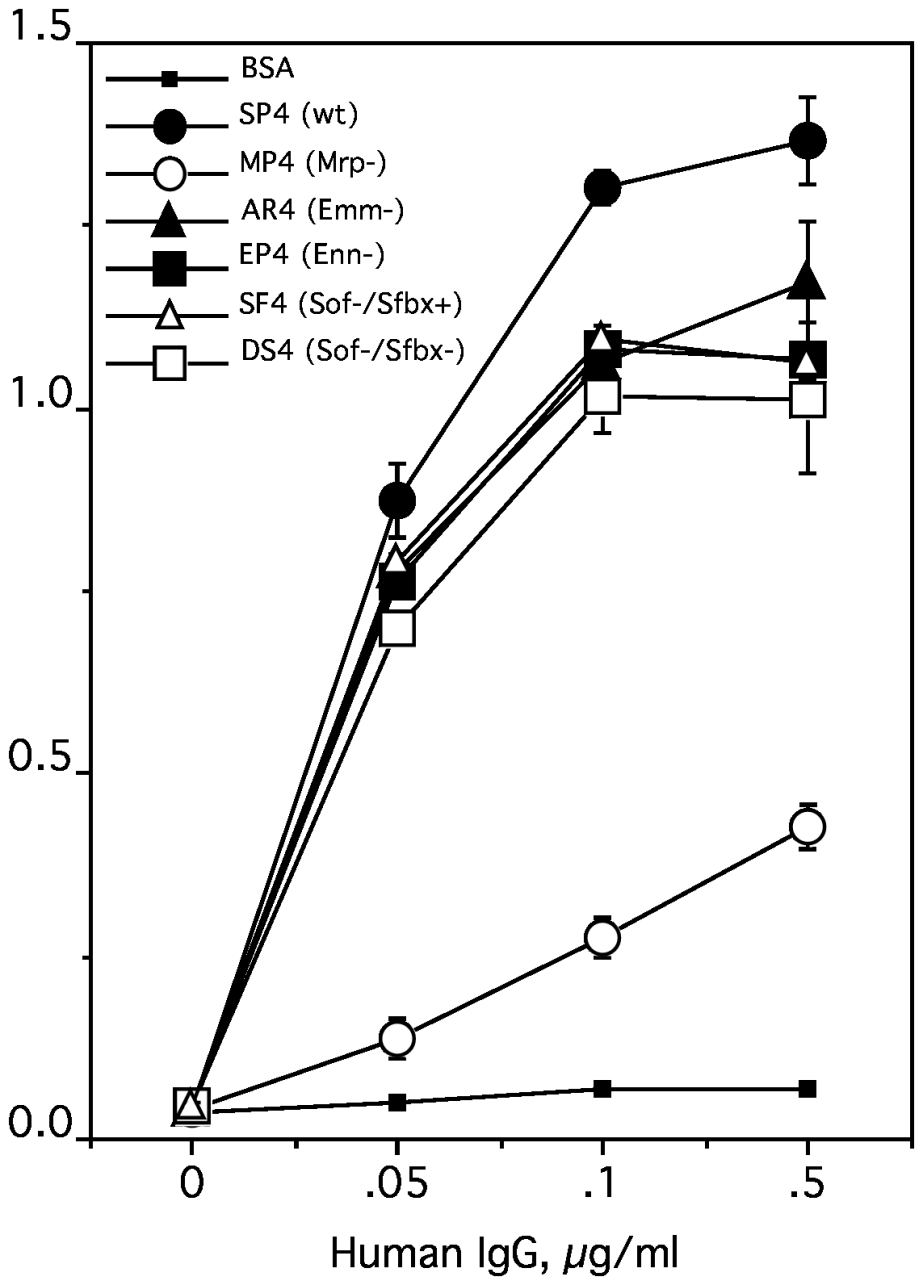
Mrp is the major IgG-binding protein in M type 4 *S. pyogenes*. Microtiter wells were coated with SP4, the wild type strain of M type 4 *S. pyogenes*, and the indicated mutants of SP4. The wells were then reacted with various concentrations of peroxidase-conjugated, human IgG. After addition of substrate the binding was determined by measuring the absorbance at 650 nm. BSA served as a negative control. Experiments were done in quadruplicate and the mean ± standard deviation is shown.

To evaluate the role of Mrp in the binding of IgG by *S. pyogenes*, the binding of human IgG to the parental, wild type strain SP4 was compared to that of the Mrp-negative mutant MP4 and various other mutants of SP4 defective in expressing other, major proteins on the surface of *S. pyogenes*. The wild type strain SP4 bound human IgG in a dose-related fashion (Figure 2). Inactivation of Mrp reduced IgG binding by ~70%, whereas inactivation of Emm, Enn, Sof, or Sfbx had only minor effects on binding. That inactivation of Mrp did not completely reduce IgG binding was not surprising as other streptococcal surface components such as Emm also bind IgG [7]. However, these findings clearly indicate that Mrp is the major IgG-binding protein on SP4.

### Selective binding of human IgG by Mrp

**Figure 3:**
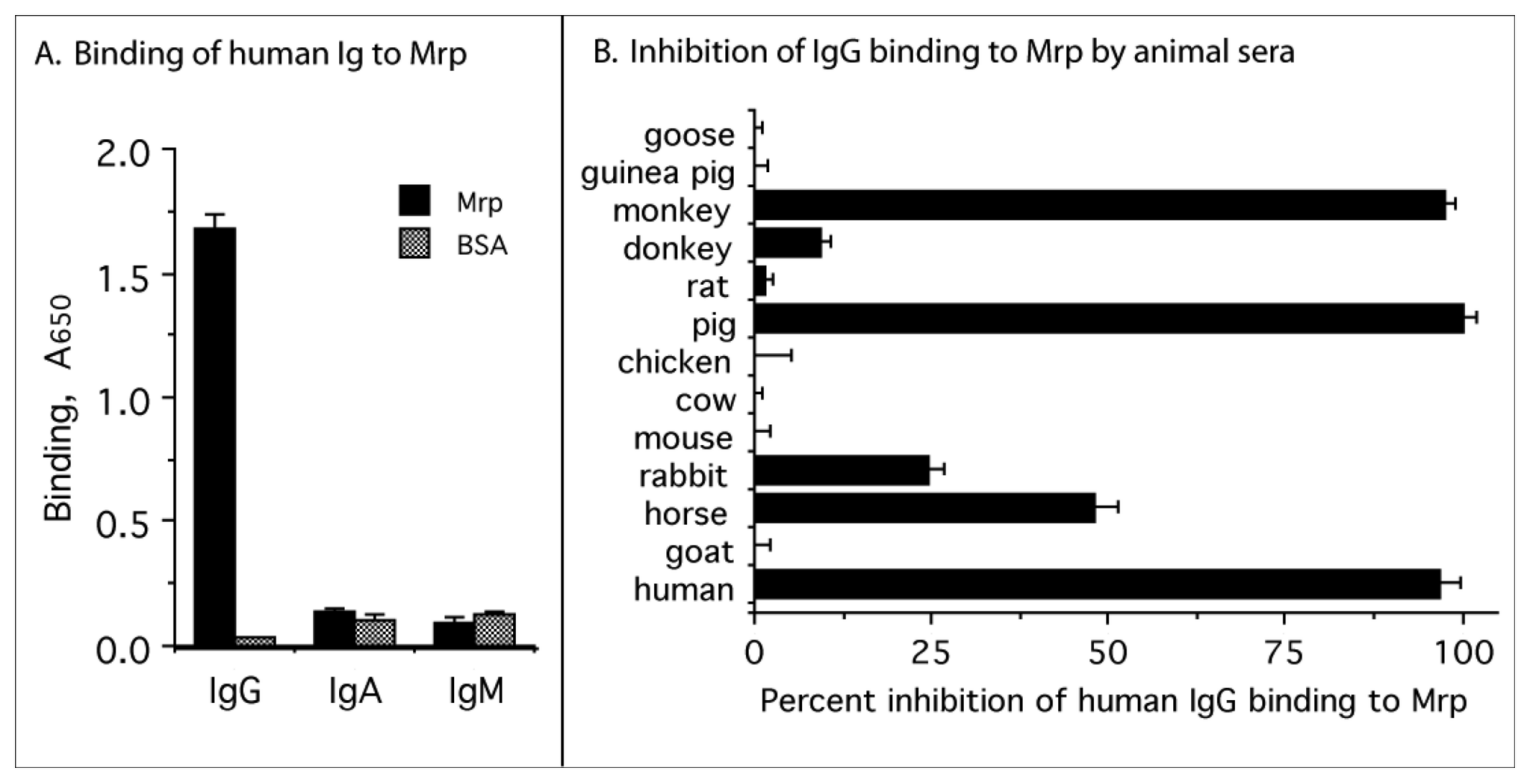
Binding of immunoglobulins to Mrp and inhibition of binding by animal sera. A. Binding of peroxidase-labeled human IgG, human IgM or human IgA to microtiter wells coated with rMrp. B. Inhibition of binding of peroxidase-labeled human IgG to rMrp by a 10% solution of serum from the indicated animals. Experiments were done in triplicate and the mean ± standard deviation is shown.

To determine if Mrp4 selectively binds to human IgG, IgA, or IgM, microtiter wells were coated with rMrp and reacted with peroxidase-labeled human IgG, IgA, or IgM. Mrp4 strongly bound to human IgG but not to human IgM, or IgA (Figure 3A). These findings are consistent with those of others who also found that Mrp selectively bound to human IgG [7,16,18,21]. 

To examine the potential for host specificity in binding, various animal sera were tested for their ability to block the binding of human IgG to Mrp (Figure 3B). Human, pig and monkey sera were the best inhibitors of binding. Horse, rabbit and donkey sera partially inhibited binding. Goose, guinea pig, rat, chicken, cow, mouse and goat sera had little to no effect on binding of human IgG to Mrp. 

**Figure 4:**
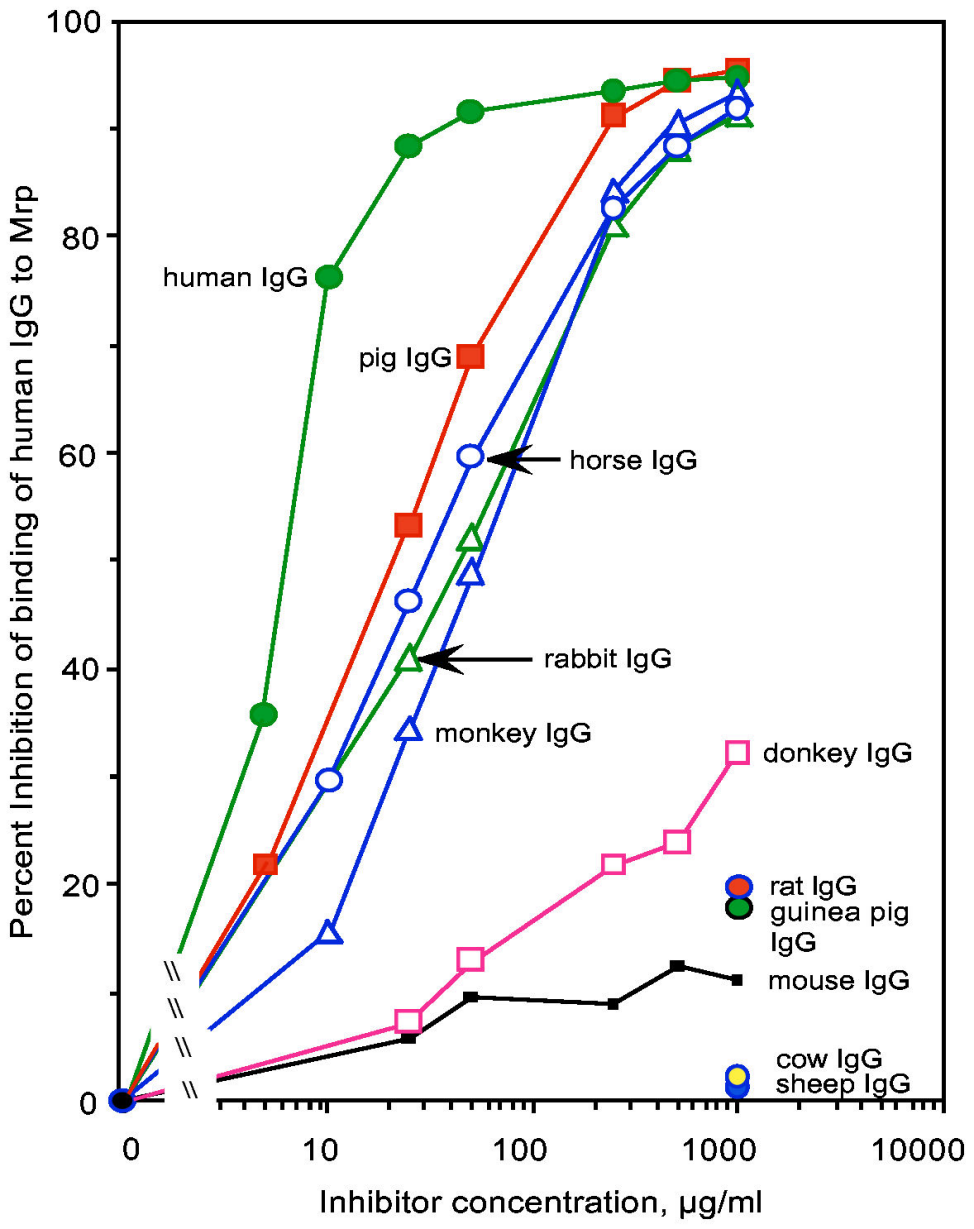
Inhibition of the binding of human IgG to Mrp by IgG from various animals. Microtiter wells were coated with rMrp and the binding of peroxidase-labeled human IgG was determined in the presence of the indicated concentrations of purified IgG from animals. Experiments were done in quadruplicate and the mean is shown.

It is possible that animal sera may contain other components that could bind to Mrp and sterically hinder interactions with IgG. Therefore, various concentrations of purified IgG from different animals were also tested for their ability to block the binding of human IgG to Mrp (Figure 4). The results essentially reflected those obtained with animal sera. For a better comparison of binding, the concentration required for 50% inhibition was calculated for each purified IgG (Table 1). Human IgG was the most effective inhibitor requiring 6 µg/ml to achieve 50% inhibition followed by pig (20 µg/ml), horse (29 µg/ml) rabbit (40 µg/ml) and monkey IgG (50 µg/ml). Thus, human IgG was > 3-fold better inhibitor than pig IgG, the one closest to human IgG in blocking binding. Mouse, rat, goat, sheep, cow, guinea pig, donkey and chicken IgG were ineffective inhibitors. 

**Table 1:** The concentration of various animal IgG required to achieve fifty percent inhibition of the binding of human IgG to Mrp4.

Origin of IgG	Concentration for 50% inhibition, µg/ml*
Human	6
Pig	20
Horse	29
Rabbit	40
Monkey	50
Mouse	>1,000
Rat	>1,000
Goat	>1,000
Sheep	>1,000
Cow	>1,000
Guinea pig	>1,000
Donkey	>1,000
Chicken	>1,000

* the 50% concentration was determined from data in Figure 4.

**Figure 5:**
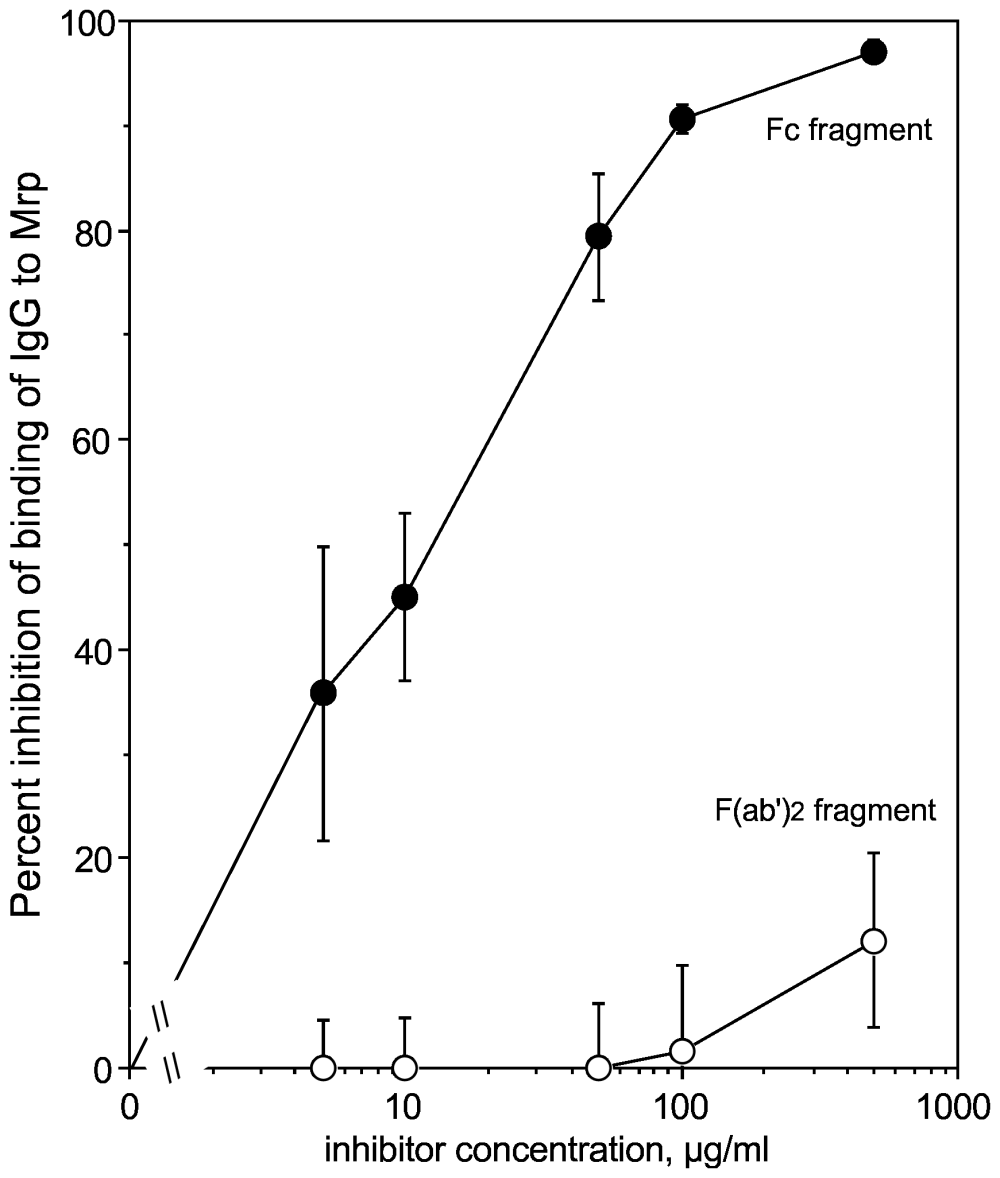
Mrp binds to the Fc region of human IgG. Various concentrations of the Fc or the F(ab’)_2_ fragment of human IgG were mixed with peroxidase-labeled human IgG and added to microtiter wells coated with Mrp and the percentage of inhibition of the binding of IgG was determined as described in Materials and Methods. Experiments were done in triplicate and the mean ± SD is shown.

Most immunoglobulin-binding proteins of bacteria bind immunoglobulins via the Fc domain. Mrp is no exception. The Fc domain of human IgG blocked the binding of human IgG to rMrp(1-328), whereas the F(ab’)_2_ fragment of human IgG had little effect on binding (Figure 5). These findings agree with those of Heath et al. [16] who found that Mrp from an M type 76 strain of *S. pyogenes* bound human IgG via its Fc domain. 

**Figure 6:**
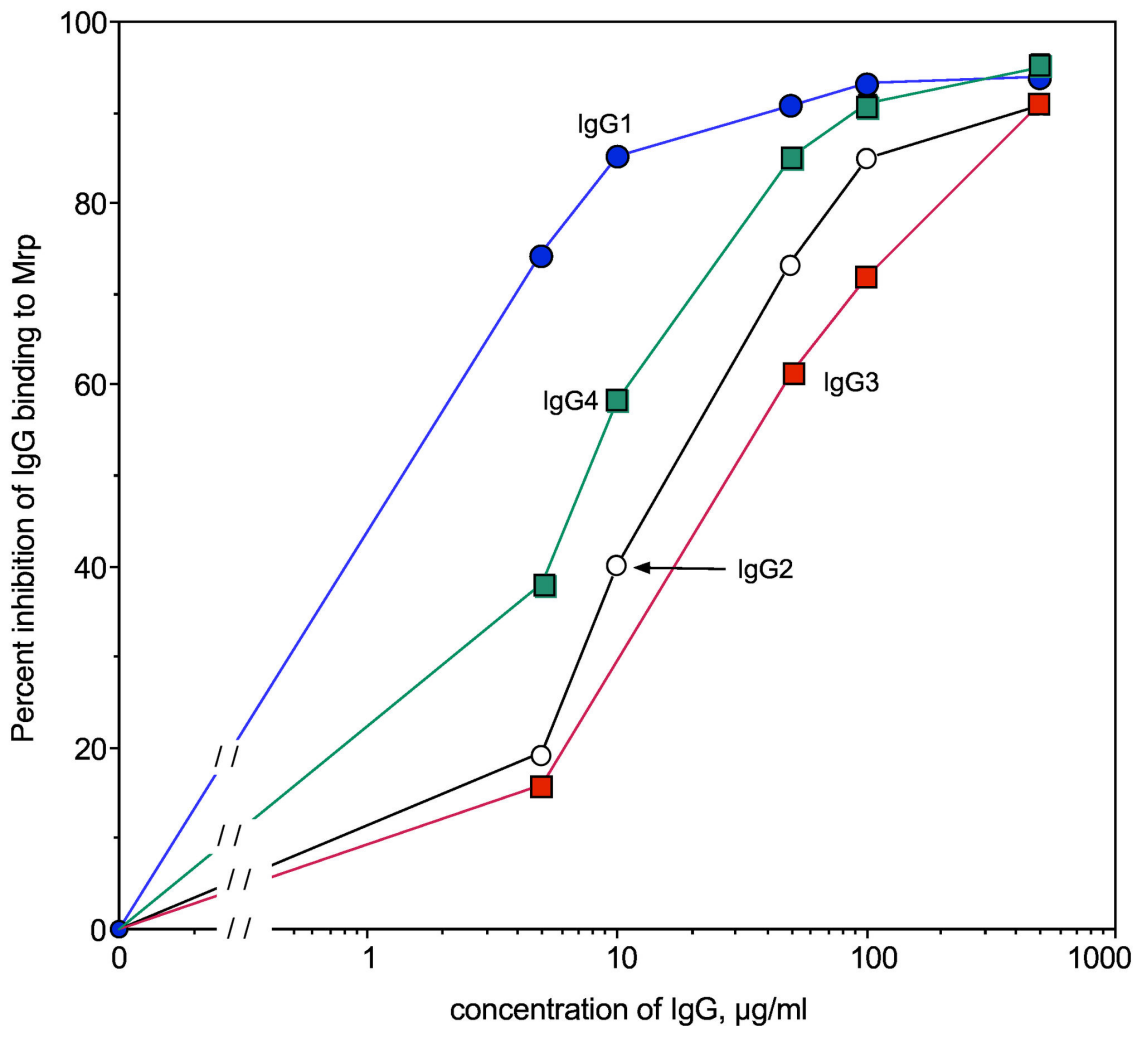
Selective binding of Mrp to subclasses of IgG. Various concentrations of IgG_1_, IgG_2_, IgG_3_ and IgG_4_ were tested for their ability to inhibit the binding of human IgG to Mrp as described in Materials and Methods. Assays were done in triplicate and the average is shown.

To further examine the binding of Mrp to human IgG, each of the subclasses was tested for its ability to competitively inhibit the binding of peroxidase-labeled human IgG to Mrp (Figure 6). The concentration required to inhibit 50% binding of peroxidase-labeled IgG was 1.2 µg/ml for IgG_1_, 15 µg/ml for IgG_2_, 30 µg/m for IgG_3_, and 7.4 µg/ml for IgG_4_. The finding that IgG_3_ was the least effective inhibitor was not unexpected because of previous reports that it bound poorly or not at all to Mrp [7,12,22]. 

### Mapping of the IgG-binding domain of Mrp

**Figure 7:**
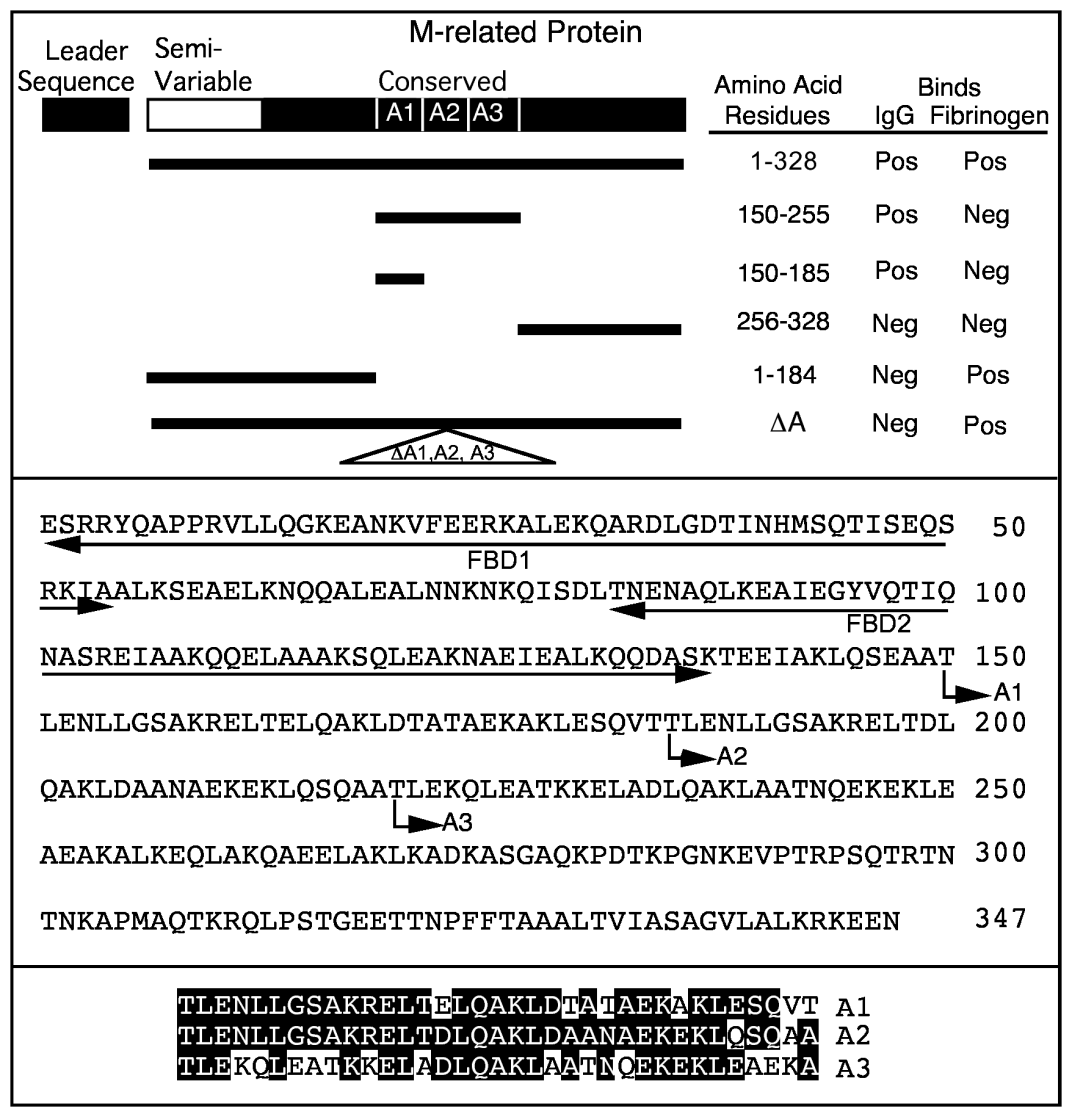
Mapping of the IgG-binding domain of Mrp. Top panel. Recombinant proteins spanning the indicated amino acid residues of Mrp were tested for binding human IgG or human fibrinogen by ELISA and the results are summarized here. Also tested was rMrpΔA, in which the DNA encoding the A-repeats were deleted in-frame as indicated by the triangle. Middle panel. The predicted sequence of the mature form of Mrp4 (leader sequence removed) is shown and the underlined sequences indicate the location of the two fibrinogen-binding domains (FBD1 and FBD2). Arrows indicate the beginning of each of the A-repeats. Bottom panel. A comparison of the sequences of the A-repeats. White letters on black background indicate amino acids that are conserved in the repeats.

**Figure 8:**
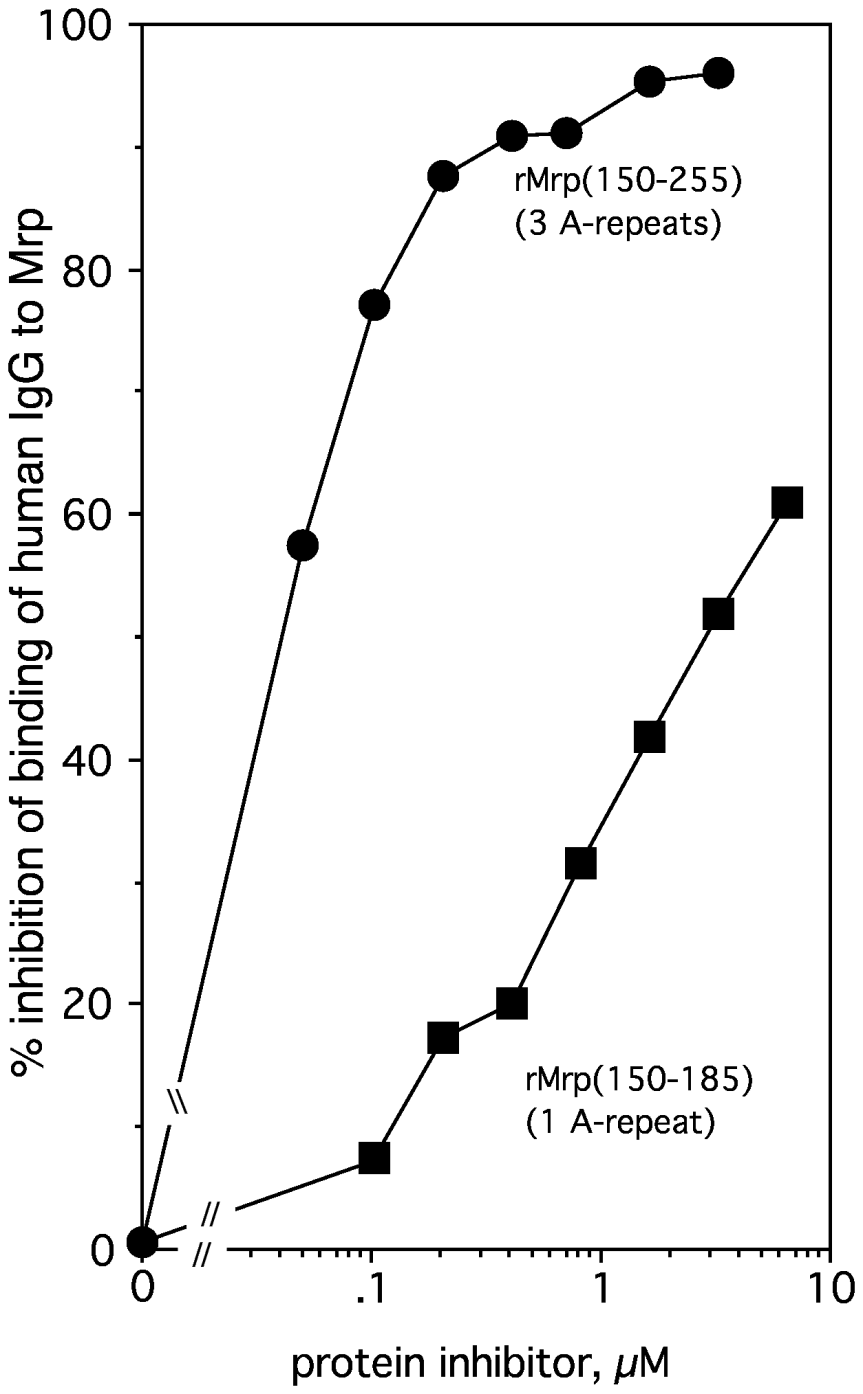
Peptide with multiple A-repeats is a better inhibitor of IgG binding to Mrp. The indicated concentrations of rMrp(150-185) (A1, a single A-repeat) or rMrp(150-255) (A3, three A repeats) were mixed with peroxidase-labeled, human IgG and the binding to microtiter wells coated with rMrp was determined as described in Materials and Methods. Assays were done in triplicate and the average is shown.

 To localize the IgG-binding domain of Mrp, various recombinant peptides of Mrp were constructed, purified and tested for binding IgG and fibrinogen by ELISA. A summary of the results is shown in Figure 7 (top panel). As expected, full-length rMrp(1-328) bound both human IgG and human fibrinogen. Both rMrp(150-185), which contains a single A-repeat, and rMrp(150-255) which contains three A-repeats, bound IgG but did not bind fibrinogen. Both rMrp(150-185) and rMrp(150-255) blocked the binding of human IgG to Mrp, but rMrp(150-255) was ~40-fold more effective inhibitor than rMrp(150-185) (Figure 8).

 The above results indicate that the A-repeat region of Mrp4 contains the IgG-binding domain and is consistent with the results of Heath et al. [16] who found that human IgG binds to the A-repeats of Mrp from an M type 76 strain of *S. pyogenes*. To confirm that the A-repeats are the only IgG-binding domain within Mrp, a recombinant protein was constructed in which the DNA encoding the A-repeat region was deleted in-frame and tested for binding human IgG. This construct failed to bind human IgG but it still retained the ability to bind fibrinogen (Figure 7, top panel). Previous work [11] indicated that there are two fibrinogen-binding domains in the N-terminus of Mrp4 as illustrated in the middle panel of Figure 7. Our data indicate that the binding domains for IgG and fibrinogen are separate and distinct. Supporting this concept is the finding that purified, human fibrinogen did not block the binding of IgG to Mrp (data not shown). 

### Role of the A-repeats of Mrp in the growth of *S. pyogenes* in human blood

**Figure 9:**
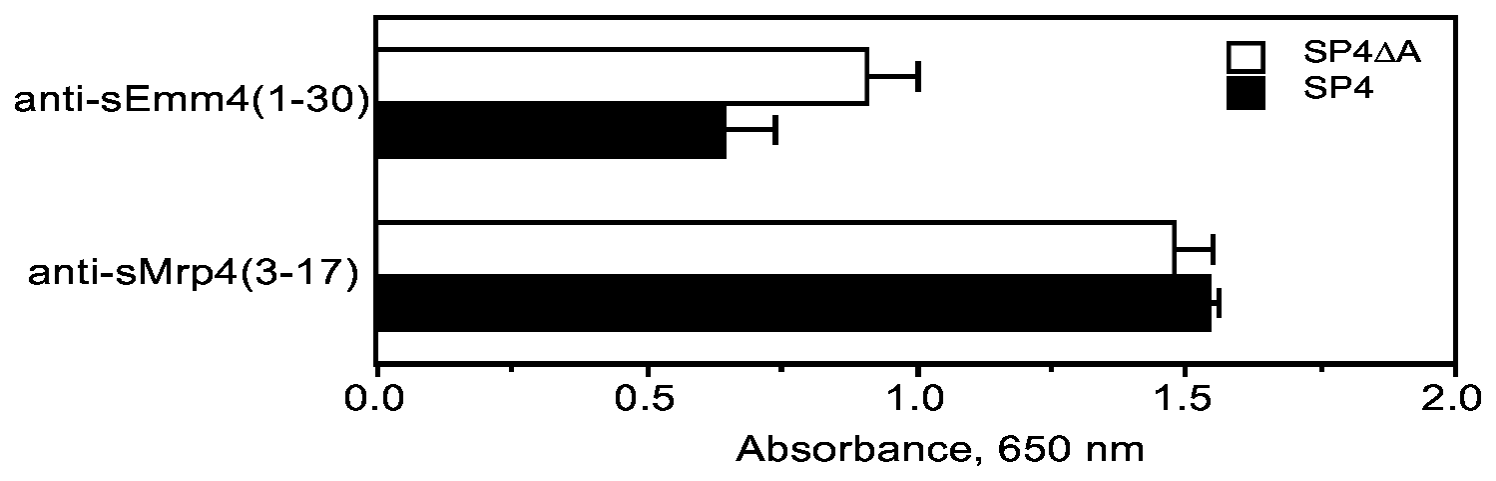
Expression of Emm and Mrp in SP4 and SP4ΔA. Microtiter wells were coated with the wild type strain SP4 and its mutant SP4ΔA and reacted with antisera against a synthetic peptide of Emm4 or Mrp4 as described in Materials and Methods. Assays were done in triplicate and the mean ± SD is shown.

The finding that inactivation of Mrp dramatically reduced IgG binding provided clear evidence that Mrp is a major IgG-binding protein in *S. pyogenes* that express Mrp. However, inactivation of Mrp not only reduces IgG binding by *S. pyogenes*, but it also reduces the binding of fibrinogen to *S. pyogenes*, which is involved in resistance to phagocytosis [5]. Therefore, evaluation of the role of IgG binding to Mrp in the growth of *S. pyogenes* in human blood must be done without altering the binding of fibrinogen to Mrp on the surface of the streptococci. To accomplish this a mutant, SP4ΔA, was engineered that expresses Mrp with an in-frame deletion of the DNA encoding the A-repeats of Mrp. We compared the levels of expression of Emm4 and Mrp4 of this mutant to that of wild type to ensure that the expression of these key proteins was not decreased by the introduction of this mutation. The expression of Mrp in SP4ΔA was virtually identical to that of its wild type parent, SP4 (Figure 9). There was a slight increase in reactivity of anti-sEmm4(1-30) serum with SP4ΔA as compared to that of SP4. The reason for this slight increase is not clear but it has been suggested that alterations of one surface protein may provide enhanced access of antibodies to another surface protein [5]. However, it is clear that there was no significant reduction in the expression of Mrp4 or Emm4 on the surface of the streptococci due to this mutation. 

**Figure 10:**
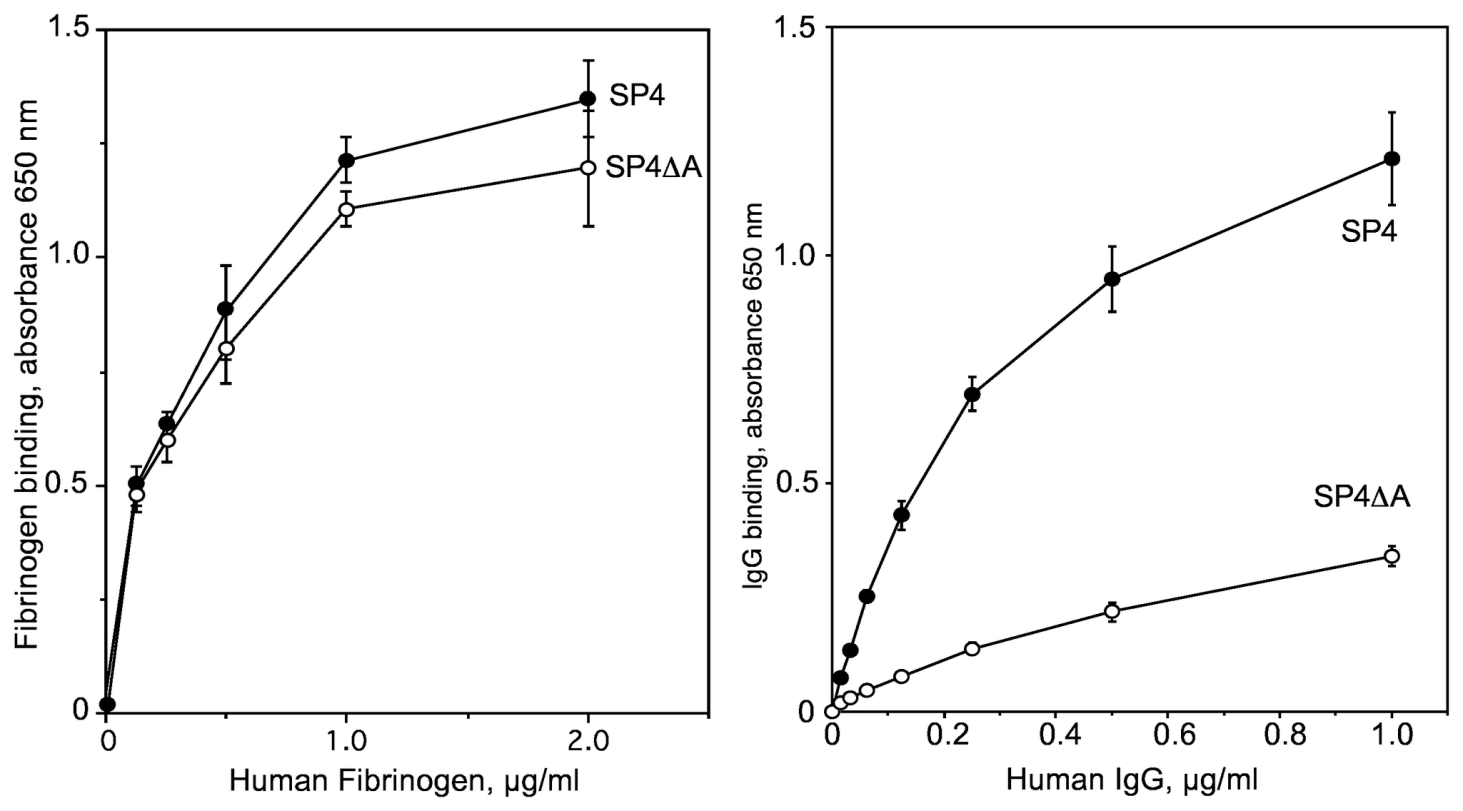
Role of the A-repeats of Mrp in the binding of human IgG and fibrinogen by *S. pyogenes*. Microtiter wells were coated with wild type M4 *S. pyogenes* (SP4) and its mutant SP4ΔA. The wells were then incubated with indicated concentrations of human fibrinogen (left panel) or human IgG (right panel). The amount bound was determined as described in Materials and Methods. Assays were done in triplicate and the mean ± SD is shown.

Next, it was critical to determine if mutant SP4ΔA that did not bind IgG would still bind fibrinogen. The binding of fibrinogen by SP4 and SP4ΔA was virtually identical, but there was a dramatic reduction in the binding of human IgG by SP4ΔA when compared to SP4 (Figure 10). These data clearly indicate that the A-repeat region of Mrp is the major IgG-binding receptor on the surface of SP4. Furthermore, the finding that fibrinogen binding of SP4ΔA was similar to that of SP4 provided additional evidence that the expression levels of Mrp was not altered by the in-frame deletion of the A-repeats, because Mrp is the major fibrinogen-binding protein in SP4 [5].

To evaluate the effect of this mutation on resistance to phagocytosis, the growth of SP4 and SP4ΔA in human blood was compared (Table 2). The multiplication factor of SP4 was 133.7 ± 16, whereas the multiplication factor of SP4ΔA was 27.1 ± -.7. This indicates that the loss of the A-repeats within Mrp reduced growth of *S. pyogenes* in human blood by ~80%. 

**Table 2:** Effect of deletion of A repeats of Mrp on growth of *S. pyogenes* in human blood.

	Strain	Inoculum	Total CFU	MF^a^
Exp 1	SP4	138	17,300	125.3
	SP4∆A	254	6,750	26.5
Exp 2	SP4	178	22,100	124.2
	SP4∆A	258	7,200	27.9
Exp 3	Sp4	182	27,600	151.6
	SP4∆A	284	7,650	26.9
Mean MF ± sd^b^	SP4 SP4∆A			133.7 ± 16 27.1 ± 0.7

The indicated inoculum of wild type *S. pyogenes* (SP4) and its mutant SP4∆A was added to human blood, rotated for 3 hours at 37°C and the total number of CFU determined.

a MF = multiplication factor, calculated by dividing the total CFU by the inoculum.

b Means are significantly different, p = 0.0003.

## Discussion

Resistance to phagocytosis is a key mechanism contributing to the virulence of *S. pyogenes*. Previous work indicated that Mrp contributes to this resistance by binding fibrinogen and preventing complement deposition [5]. Purified Mrp has also been shown to bind human IgG, but it was not known if Mrp is the major surface protein that binds IgG or if this binding has any role in resistance to phagocytosis in human blood. In this report, we present our findings indicating that the Fc-mediated binding of human IgG to the A-repeats of Mrp does confer resistance to phagocytosis and enhances growth of *S. pyogenes* in human blood. These findings include: (i) inactivation of Mrp resulted in a dramatic decrease in the binding of human IgG by *S. pyogenes*; (ii) purified, recombinant peptides expressing the A-repeats of Mrp bound human IgG whereas other recombinant peptides of Mrp did not; (iii) Mrp bound to the Fc domain of human IgG; (iv) SP4ΔA, a mutant expressing Mrp with an in-frame deletion of A-repeats, exhibited reduced binding of human IgG but still bound fibrinogen equal to SP4; (v) growth of SP4ΔA in human blood was reduced by 80% compared to its wild type parent. 

These results indicate that Mrp is a key virulence factor that contributes to resistance to phagocytosis not only by binding fibrinogen, but also by binding human IgG. The clinical relevance of this finding is that Mrp is widely expressed by strains of *S. pyogenes* causing infections: ~75% of serotypes found during a surveillance by the Center for Disease Control during 1995-2001 contain the gene for Mrp. 

Unlike M proteins that exhibit high variability within their N-termini, Mrp’s are only semi-variable in their N-termini. Analysis of Mrp sequences indicated that there are three major groups of Mrp based on variability within their N-termini and within each major group the N-termini are highly conserved [11]. In addition, the C-terminal regions of Mrp are highly conserved. Thus, the overall similarity among Mrp from different serotypes is quite high and ranges from 80 to 98% [11]. Therefore, functions that are associated with domains within the conserved region of one Mrp are likely to be found in all Mrp’s. Supporting this notion is the finding that all *mrp* sequenced to date contain the A-repeat region, indicating that the ability to bind IgG is likely to be a common trait among strains expressing Mrp [11]. Further support for this concept is the finding that human IgG binds to Mrp’s representing all three groups of Mrp [7,16,17].

The A-repeats of Mrp appear to contain unique sequences that bind IgG. The IgG-binding domain of Mrp does not have any significant degree of similarity to the IgG-binding domains of M proteins and protein H, as described by Pack et al. [23] or to the IgA-binding domains of M proteins and M-like proteins described by others [9,13,24].

Our competitive inhibition data suggest that the A-repeats of Mrp preferentially bind IgG subclasses in the order of IgG_1_>IgG_4_>IgG_2_>IgG_3_. Mrp bound IgG_1_ 6-fold better than IgG_4,_ 12-fold better than IgG_2_ and 25-fold better than IgG_3_. Previous investigations using western blot analyses suggested that Mrp binds human IgG_1_, IgG_2_, and IgG_4_ but not IgG_3_ [7,12], whereas another investigation using Ouchterlony immunodiffusion analysis indicated that Mrp bound all of the subclasses, but bound IgG_3_ weakest [22]. In prior analyses, where the binding of IgG_3_ was not detected, only one concentration was used in western blots, whereas we used a competitive inhibition assay with low to high concentrations of IgG, which may detect weaker binding. 

The preferential binding of human IgG to Mrp may have a role in host specificity. GAS infections are almost entirely restricted to the human host and the findings that Mrp selectively binds human IgG and that this binding enables the GAS to resist phagocytosis in human blood, suggest that this binding may be a factor contributing to this host restriction. In this regard, it is interesting to note that the mouse model is widely used for testing the virulence of GAS, yet mouse IgG does not bind to Mrp. Future work should examine whether a transgenic mouse expressing human IgG would be more susceptible to GAS infections.

The molecular mechanism(s) whereby IgG-Mrp interactions contribute to resistance to phagocytosis is not entirely clear. One mechanism may be that the acquisition of IgG on the surface of the streptococci makes the surface of the bacteria appear more like the host and thereby, reduces the ability of the host to detect the bacteria. It is not suggested that IgG binding alone may accomplish this as GAS bind a large number of blood proteins and become coated with these host proteins. However, the binding of IgG to Mrp may contribute to this process. One possible consequence of this coating is the diminished deposition of complement onto the streptococcal surface. It remains to be determined if Mrp-IgG interactions have any effect on complement deposition.

There are other potential mechanisms that may come into play in the presence of antibodies directed against surface antigens of *S. pyogenes*. The binding of IgG via its Fc may prevent antibodies from binding via its antigen specific Fab domains and thereby reduce opsonization. IgG bound via its Fc domain to Mrp would not be able to bind to Fc receptors on phagocytic cells. In this regard, the streptococcal, IgG-cleaving enzyme, IdeS, was found to preferentially degrade IgG bound to streptococcal proteins via Fab interactions compared to IgG bound via Fc interactions [25]. Thus, Mrp could act in concert with IdeS to prevent phagocytosis. IdeS would degrade IgG bound to surface antigens via its Fab while IgG bound to Mrp by the Fc regions would not be degraded by IdeS, but would prevent interactions with Fc receptors on phagocytic cells. 

A similar mechanism for the IgA-binding proteins Emm22 (also termed Sir22) and Emm4 (also termed Arp4) has been proposed by Woof [26], where these IgA-binding proteins were found to inhibit the binding of IgA to FcαR. Emm4 was also found to inhibit FcαR triggering of the respiratory burst in neutrophils [13]. It was suggested that when IgA binds to specific antigens on the surface of *S. pyogenes*, the Fc domain of the bound IgA molecules then interacts with these Emm proteins and thereby, interferes with binding to their Fc receptors on phagocytes. That IgA binding to Emm22 is involved in resistance to phagocytosis was shown by Carlsson et al. [6] who found that an in-frame deletion of the IgA-binding domain in Emm22 reduced growth of *S. pyogenes* in human blood. While the Mrp-binding domain has been determined to be in the Fc domain of IgG, it is not known if this binding site overlaps with that for Fcγreceptors on IgG. Thus, it is not known if Mrp will compete with Fcγreceptors for binding the Fc regions of IgG. Further work is needed to determine the molecular mechanism(s) whereby Mrp confers resistance to phagocytosis in human blood.

It was recently suggested by Nordenfelt et al. [27] that the Fc-mediated binding of IgG by *S. pyogenes* does not provide protection against phagocytosis in blood. This was based, in part, on the finding that expression of protein H in an M1 strain of *S. pyogenes* failed to protect against phagocytosis when the streptococci were coated with antibodies that bound via antigen-specific Fab domain of IgG. However, it is well known that opsonic antibodies specific for certain streptococcal surface antigens can promote phagocytosis and killing of GAS, thereby overcoming the ability of GAS to resist phagocytosis and multiply in human blood [28]. One of the major differences between our experiments and those of Nordenfelt et al. is that we utilized whole blood from donors who had been screened for the absence of opsonic antibodies, whereas in their study the IgG preparations used in phagocytosis assays appeared to contain opsonizing antibodies, because their IgG preparations promoted phagocytosis of GAS and by definition IgG that promotes phagocytosis is considered opsonic. Another, important difference between these two studies is that the M1 strain used by Nordenfelt et al. does not contain the gene for *mrp*, whereas Mrp is clearly expressed in the M type 4 strain used in our study. Although the Emm1 strain does bind IgG, this binding is mediated by protein H and Emm1 and it may be that this binding will not have the same impact on GAS growth in blood as that found for IgG binding to Mrp. 

In summary, the A-repeats of Mrp contribute to virulence of group A streptococci by binding IgG and enhancing resistance to phagocytosis in human blood. Its preferential binding of human IgG may contribute to the host specificity of this organism. Because Mrp is a virulence factor, its potential as a vaccine candidate should be investigated to determine if antibodies directed against the A-repeats are opsonic. Such antibodies might be effective not only by directly opsonizing the bacteria but also by blocking interactions between the A-repeats of Mrp and the Fc domain of IgG.

## References

[1] Carapetis JR, Steer AC, Mulholland EK, Weber M (2005) The global burden of group A streptococcal diseases. Lancet Infect Dis 5: 685-694. doi:10.1016/S1473-3099(05)70267-X. PubMed: 16253886. 16253886

[2] Sandin C, Carlsson F, Lindahl G (2006) Binding of human plasma proteins to Streptococcus pyogenes M protein determines the location of opsonic and non-opsonic epitopes. Mol Microbiol 59: 20-30. doi:10.1111/j.1365-2958.2005.04913.x. PubMed: 16359315. 16359315

[3] Courtney HS, Liu S, Dale JB, Hasty DL (1997) Conversion of M serotype 24 of Streptococcus pyogenes to M serotypes 5 and 18: effect on resistance to phagocytosis and adhesion to host cells. Infect Immun 65: 2472-2474. PubMed: 9169794. 9169794 10.1128/iai.65.6.2472-2474.1997 PMC175346

[4] Sanderson-Smith M, Batzloff M, Sriprakash KS, Dowton M, Ranson M et al. (2006) Divergence in the plasminogen-binding group a streptococcal M protein family: functional conservation of binding site and potential role for immune selection of variants. J Biol Chem 281: 3217-3226. PubMed: 16319056. 16319056 10.1074/jbc.M508758200

[5] Courtney HS, Hasty DL, Dale JB (2006) Anti-phagocytic mechanisms of Streptococcus pyogenes: binding of fibrinogen to M-related protein. Mol Microbiol 59: 936-947. doi:10.1111/j.1365-2958.2005.04977.x. PubMed: 16420362. 16420362

[6] Carlsson F, Berggård K, Stålhammar-Carlemalm M, Lindahl G (2003) Evasion of phagocytosis through cooperation between two ligand-binding regions in Streptococcus pyogenes M protein. J Exp Med 198: 1057-1068. doi:10.1084/jem.20030543. PubMed: 14517274. 14517274 PMC2194224

[7] Podbielski A, Schnitzler N, Beyhs P, Boyle MD (1996) M-related protein (Mrp) contributes to group A streptococcal resistance to phagocytosis by human granulocytes. Mol Microbiol 19: 429-441. doi:10.1046/j.1365-2958.1996.377910.x. PubMed: 8830235. 8830235

[8] Svensson MD, Sjöbring U, Luo F, Bessen DE (2002) Roles of the plasminogen activator streptokinase and the plasminogen-associated M protein in an experimental model for streptococcal impetigo. Microbiology 148: 3933-3945. PubMed: 12480897. 12480897 10.1099/00221287-148-12-3933

[9] Bessen DE (1994) Localization of immunoglobulin A-binding sites within M or M-like proteins of group A streptococci. Infect Immun 62: 1968-1974. PubMed: 8168964. 8168964 10.1128/iai.62.5.1968-1974.1994 PMC186455

[10] Berggård K, Johnsson E, Morfeldt E, Persson J, Stålhammar-Carlemalm M et al. (2001) Binding of human C4BP to the hypervariable region of M protein: a molecular mechanism of phagocytosis resistance in Streptococcus pyogenes. Mol Microbiol 42: 539-551. doi:10.1046/j.1365-2958.2001.02664.x. PubMed: 11703674. 11703674

[11] Li Y, Courtney HS (2011) Promotion of phagocytosis of Streptococcus pyogenes in human blood by a fibrinogen-binding peptide. Microbes Infect 13: 413-418. doi:10.1016/j.micinf.2010.12.008. PubMed: 21241819. 21241819

[12] Pack TD, Boyle MD (1995) Characterization of a type II'o group A streptococcal immunoglobulin-binding protein. Mol Immunol 32: 1235-1243. doi:10.1016/0161-5890(95)00074-7. PubMed: 8559148. 8559148

[13] Pleass RJ, Areschoug T, Lindahl G, Woof JM (2001) Streptococcal IgA-binding proteins bind in the Calpha 2-Calpha 3 interdomain region and inhibit binding of IgA to human CD89. J Biol Chem 276: 8197-8204. doi:10.1074/jbc.M009396200. PubMed: 11096107. 11096107

[14] Sanderson-Smith ML, Dowton M, Ranson M, Walker MJ (2007) The plasminogen-binding group A streptococcal M protein-related protein Prp binds plasminogen via arginine and histidine residues. J Bacteriol 189: 1435-1440. doi:10.1128/JB.01218-06. PubMed: 17012384. 17012384 PMC1797364

[15] Sun H, Ringdahl U, Homeister JW, Fay WP, Engleberg NC et al. (2004) Plasminogen is a critical host pathogenicity factor for group A streptococcal infection. Science 305: 1283-1286. doi:10.1126/science.1101245. PubMed: 15333838. 15333838

[16] Heath DG, Boyle MD, Cleary PP (1990) Isolated DNA repeat region from fcrA76, the Fc-binding protein gene from an M-type 76 strain of group A streptococci, encodes a protein with Fc-binding activity. Mol Microbiol 4: 2071-2079. doi:10.1111/j.1365-2958.1990.tb00567.x. PubMed: 2089220. 2089220

[17] Stenberg L, O'Toole P, Lindahl G (1992) Many group A streptococcal strains express two different immunoglobulin-binding proteins, encoded by closely linked genes: characterization of the proteins expressed by four strains of different M-type. Mol Microbiol 6: 1185-1194. doi:10.1111/j.1365-2958.1992.tb01557.x. PubMed: 1588817. 1588817

[18] O'Toole PW, Stenberg L, Rissler M, Lindahl G (1992) Two major classes in the M protein family in group A streptococci. Proc Natl Acad Sci U S A 89: 8661-8665. doi:10.1073/pnas.89.18.8661. PubMed: 1528877. 1528877 PMC49980

[19] Bayer EA, Skutelsky E, Wilchek M (1979) The avidin-biotin complex in affinity cytochemistry. Methods Enzymol 62: 308-315. doi:10.1016/0076-6879(79)62235-8. PubMed: 440114. 440114

[20] Maguin E, Prévost H, Ehrlich SD, Gruss A (1996) Efficient insertional mutagenesis in lactococci and other gram-positive bacteria. J Bacteriol 178: 931-935. PubMed: 8550537. 8550537 10.1128/jb.178.3.931-935.1996 PMC177749

[21] Krebs B, Kaufhold A, Boyle MD, Podbielski A (1996) Different alleles of the fcrA/mrp gene of Streptococcus pyogenes encode M-related proteins exhibiting an identical immunoglobulin-binding pattern. Med Microbiol Immunol (Berl) 185: 39-47. doi:10.1007/s004300050013. PubMed: 8803952. 8803952

[22] Heath DG, Cleary PP (1987) Cloning and expression of the gene for an immunoglobulin G Fc receptor protein from a group A streptococcus. Infect Immun 55: 1233-1238. PubMed: 2952595. 2952595 10.1128/iai.55.5.1233-1238.1987 PMC260495

[23] Pack TD, Podbielski A, Boyle MD (1996) Identification of an amino acid signature sequence predictive of protein G-inhibitable IgG3-binding activity in group-A streptococcal IgG-binding proteins. Gene 171: 65-70. doi:10.1016/0378-1119(96)00102-3. PubMed: 8675032. 8675032

[24] Johnsson E, Andersson G, Lindahl G, Hedén LO (1994) Identification of the IgA-binding region in streptococcal protein Arp. J Immunol 153: 3557-3564. PubMed: 7930578. 7930578

[25] Su YF, Chuang WJ, Wang SM, Chen WY, Chiang-Ni C et al. (2011) The deficient cleavage of M protein-bound IgG by IdeS: insight into the escape of Streptococcus pyogenes from antibody-mediated immunity. Mol Immunol 49: 134-142. doi:10.1016/j.molimm.2011.08.002. PubMed: 21925735. 21925735

[26] Woof JM (2002) The human IgA-Fc alpha receptor interaction and its blockade by streptococcal IgA-binding proteins. Biochem Soc Trans 30: 491-494. PubMed: 12196121. 12196121 10.1042/bst0300491

[27] Nordenfelt P, Waldemarson S, Linder A, Mörgelin M, Karlsson C et al. (2012) Antibody orientation at bacterial surfaces is related to invasive infection. J Exp Med 209: 2367-2381. doi:10.1084/jem.20120325. PubMed: 23230002. 23230002 PMC3526361

[28] Lancefield RC (1962) Current knowledge of type-specific M antigens of group A streptococci. J Immunol 89: 307-313. PubMed: 14461914. 14461914

[29] Kalia A, Bessen DE (2004) Natural selection and evolution of streptococcal virulence genes involved in tissue-specific adaptations. J Bacteriol 186: 110-121. doi:10.1128/JB.186.1.110-121.2004. PubMed: 14679231. 14679231 PMC303441

[30] Bessen DE, Sotir CM, Readdy TL, Hollingshead SK (1996) Genetic correlates of throat and skin isolates of group A streptococci. J Infect Dis 173: 896-900. doi:10.1093/infdis/173.4.896. PubMed: 8603968. 8603968

[31] Courtney HS, Pownall HJ (2010) The structure and function of serum opacity factor: a unique streptococcal virulence determinant that targets high-density lipoproteins. J Biomed Biotechnol 2010: 956071 PubMed: 20671930 10.1155/2010/956071 PMC2910554 20671930

[32] Courtney HS, Ofek I, Penfound T, Nizet V, Pence MA et al. (2009) Relationship between expression of the family of M proteins and lipoteichoic acid to hydrophobicity and biofilm formation in Streptococcus pyogenes. PLOS ONE 4: e4166. doi:10.1371/journal.pone.0004166. PubMed: 19132104. 19132104 PMC2613554

